# Towards an information-centric architecture framework for health information logistics: a design science research study

**DOI:** 10.1186/s12911-026-03642-7

**Published:** 2026-06-26

**Authors:** Øivind Skeidsvoll Solvang, Francis Odeh, Conceição Granja, Terje Solvoll

**Affiliations:** 1 Department of Strategic ICT, Helse Vest IKT, Gimlebakken 1, Bergen, 5052 Norway; 2 Norwegian Centre for E-health Research, University Hospital of North Norway, Tromsø, Norway; 3 Faculty of Nursing and Health Sciences, Nord University, Bodø, Norway; 4 Department of Health and Social Care, Municipality of Bodø, Bodø, Norway; 5 Department of Community Medicine, UiT – The Arctic University of Norway, Tromsø, Norway

**Keywords:** Integrated care, Health information exchange, Health informatics, Electronic health records, Interoperability, Information governance, Data management, Health information quality, Virtual EHR, Design science research

## Abstract

**Background:**

Integrated care depends on timely awareness of parallel treatment activities and safe access to distributed patient records. However, multimorbid patients often navigate complex, non-linear pathways across care levels, resulting in information fragmentation that compromises decision-making, safety, and coordination. Existing electronic health record systems and interoperability initiatives offer limited support for pathway-oriented information logistics, particularly with respect to governance, provenance, and the integration of the patient’s voice. This study aims to design a conceptual information-centric architecture framework to address these systemic gaps in awareness and access.

**Methods:**

Applying Design Science Research (DSR), the study synthesized rigor from a scoping review with relevance derived from a longitudinal analysis of medical records from 14 multimorbid patients (1954–2024) in Northern Norway. A representative 19-year user scenario, validated by clinical expertise, together with stakeholder and root-cause analyses, informed the identification of recurrent failures in information logistics. High-level non-functional and functional requirements were iteratively derived, foregrounding Information Governance (IG), Data Management (DM), Security and Privacy, and Health Information Quality (HIQ) as essential socio-technical constraints. The resulting architecture was examined through a scenario-based demonstration and an early-stage ex-ante evaluation.

**Results:**

The study produced a prescriptive information-centric architecture framework (Meta-artifact) organized into layered, modular components that separate user interaction, pathway logic, and secure storage. Information tokens, consisting of metadata, clinical codes, and location pointers, enable governed, read-only virtual access to source records, reducing duplication and avoiding traditional replication burdens. HIQ operationalizes DM/IG by enforcing requirements for accuracy, currency, consistency, completeness, and contextual relevance across inter-organizational access. In a hospitalization–discharge scenario, the architecture provided timely notifications, governed access to distributed records, and transparent consent and audit controls, demonstrating conceptual feasibility and alignment with the identified problem and requirements.

**Conclusions:**

This study contributes early-stage prescriptive design knowledge to improve inter-organizational information logistics. By embedding IG, DM, HIQ, and Security and Privacy as structural pillars and decoupling information from application constraints, the architecture provides a governance-aligned foundation for pathway-oriented coordination, proactive awareness, and safe, on-demand virtual access. This information-centric, token-based architecture, with a modular, standards-neutral design, supports adaptability and provides a foundation for future prototyping and empirical evaluation.

**Supplementary Information:**

The online version contains supplementary material available at https://doi.org/10.1186/s12911-026-03642-7.

## Background

Accessible, usable health information at the point of care (information logistics) is essential for coordinated, high-quality healthcare across organizational boundaries [1, 2]. Yet patients suffering from multiple diseases (multimorbidity), such as the steadily rising group of individuals with combined mental and somatic health conditions, frequently undergo concurrent treatments [3, 4], increasingly navigate complex, non‑linear pathways involving multiple providers and parallel treatment activities across specialties and care levels [5–9]. In these settings, clinicians often lack awareness of care delivered elsewhere and have limited access to patients’ voices and their distributed records [10–12], resulting in uncertainty, duplication, delays, and greater information‑seeking burdens on both clinicians and patients [1, 2, 13, 14]. These challenges are magnified by the situated character of clinical work, where coordination and communication unfold within heterogeneous practices, organizational arrangements, and documentation cultures [15–18].

Despite ongoing national and international initiatives to strengthen interoperability and promote integrated care, information fragmentation persists. As health information architectures are deeply influenced by their organizational context [19], the Norwegian healthcare system, characterized by decentralized primary care and centralized specialist services, faces coordination challenges familiar to many distributed healthcare settings. In Norway, Urgent Care (UC), General Practitioner (GP), and Community Nursing Service (CNS) are organized under 357 municipalities, with GPs and UC units acting as gatekeepers to specialized services [20]. Specialized care is organized under four state-owned regional health authorities. This distributed landscape underscores the need for information logistics through information networks that can follow patients across providers, support proactive awareness, and provide safe, governed access to relevant records [2, 21–23]. Traditional Electronic Health Record (EHR) systems, largely optimized for documentation within single organizations, offer limited support for these demands [5, 22, 24].

To address these systemic limitations, the Valkyrie research project was initiated to develop a technological prototype capable of providing virtual, pathway‑oriented access to distributed patient records without requiring physical replication [3, 25]. Virtual access in this context refers to the ability to retrieve governed, context‑relevant views of source data on demand, enabling clinicians to obtain necessary details of concurrent activities elsewhere while preserving autonomy and local data ownership.

Guided by the Design Science Research (DSR) paradigm, this study aims to design a conceptual information-centric architecture framework as prescriptive design knowledge (Meta-artifact). The framework is grounded in empirical analysis of multimorbid patient trajectories, socio‑technical theory, and existing evidence on inter-organizational information logistics. By focusing on the structural and governance conditions needed for safe, high‑quality data use across organizations, the study contributes a design-oriented response to the persistent gaps in awareness and access to distributed patient records.

To this end, this research makes three primary contributions: first, it formalizes an approach that decouples information assets from application boundaries through information tokens and governed, read-only virtual access. This enables flexible, long-term Data Management (DM) and reduces synchronization risks inherent in record replication [26]; second, the framework embeds Health Information Quality (HIQ) as a governing mechanism, specifying non-functional requirements for accuracy, currency, completeness, and contextual relevance [27]. This provides a concrete means to mitigate empirically observed failures such as data duplication, untimely information logistics, and inconsistencies across organizations [26, 28]; and third, the architectural foundation introduces modules for proactive awareness of concurrent treatment activities, and a Virtual EHR for governed, on-demand access to distributed patient records. This design can support rapid clinical sense-making at the point of care and opportunities for detailed clinical review across heterogeneous, socio-technical care environments [15, 16].

Taken together, these contributions provide a rigorously grounded, standards‑neutral blueprint for improving inter‑organizational information logistics in complex care settings. The framework is intended as an early‑stage Meta‑artifact to guide future design iterations, including technical instantiation and empirical evaluation.

## Related work

Although information fragmentation remains a persistent challenge in inter-organizational care, there has been extensive effort to strengthen integration through various reforms and international standards. Coordination reforms in Norway have aimed to improve information logistics across primary and specialist services [29–31], and initiatives such as the patient-facing national portal “Helse Norge”, and multiple digital services seek to increase patient access and support cross-provider communication [32–34]. Significant attention has also been placed on data integration through widely adopted standards such as Health Level Seven (HL7), Fast Healthcare Interoperability Resources (FHIR), openEHR, Integrating the Healthcare Enterprise (IHE), the International Organization for Standardization (ISO) - ISO 13,606, and ISO 13,940 (ContSys). However, as highlighted in the literature, these standards have not been sufficient to prevent fragmentation or to support consistent patient‑centric data sharing across heterogeneous EHR platforms [12, 32–34].

Beyond standards, existing EHR integration initiatives often rely on architectures that replicate records, enable only limited cross-system queries, or rely on messaging-based workflows. The authors’ scoping review identified six distinct approaches to inter-organizational EHR access, revealing that many existing designs were ad hoc, technology-driven, or insufficiently aligned with the requirements for pathway-driven, cross-organizational information use [26]. Critically, the review found that HIQ, including accuracy, currency, and completeness [27], was the primary enabler of successful inter-organizational information exchange. Conversely, poor information quality and pervasive trust issues emerged as systemic barriers in prior solutions [26]. These findings underscore the need for architecture that embeds governance and quality constraints directly into its design and that considers the socio‑technical conditions in which information is produced and used.

The socio‑technical literature consistently emphasizes that successful health information system design must account for heterogeneity in clinical routines and coordination practices to mitigate unintended consequences [15–17, 22, 35]. Information system design is therefore not solely a technical undertaking, but also a matter of aligning components, roles, and governance mechanisms so that information can be safely and meaningfully shared and understood across contexts. Systems‑engineering principles reinforce this need for structure and modularity: layered architectures help manage complexity, modular design supports long-term flexibility and independent evolution of components, and explicit interface specifications reduce interdependencies and clarify integration boundaries [36–40]. Together, these perspectives suggest that inter-organizational information systems must be designed as systematic, modular, and governed architectures capable of supporting varied socio‑technical settings while enforcing consistent HIQ and access constraints.

In line with these insights, the present study advances a conceptual information-centric architecture that foregrounds security and privacy, DM [41], and Information Governance (IG) [42], as authoritative, vertical pillars working alongside HIQ [27]. These governing constraints are intended to mitigate the trust and quality limitations noted in earlier approaches [26], and to provide a prescriptive foundation for safe, pathway‑oriented information logistics in distributed healthcare environments.

## Methods

**Fig. 1:**
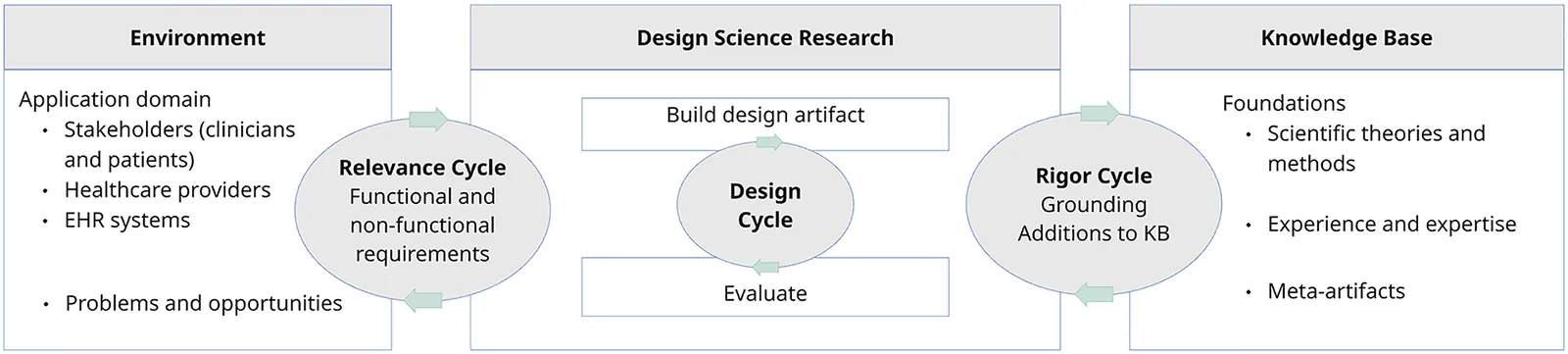
Design science research method for artifact design [46]

This study follows the Design Science Research (DSR) paradigm to construct and justify a conceptual information‑centric architecture framework (Meta‑artifact) addressing inter‑organizational information logistics in integrated care [43–46]. We adopt Hevner’s three‑cycle model (Fig. 1) to ensure relevance, rigor, and coherent artifact design [46, 47], drawing on established DSR process guidance for problem and requirement definitions, artifact construction, and early-stage evaluation [43–46].

### DSR Relevance Cycle

The Relevance Cycle establishes the problem environment and anchors the design in real‑world needs [47]. We retrospectively examined medical records from 14 multimorbid patients with combined mental and somatic conditions (1954–2024) across primary and specialist care in Northern Norway [48]. The sample included eight females (three living and five dead) and six males (one living and five dead), representing variation in age, diagnoses, and care trajectories across healthcare levels and multiple EHRs. Ten deceased patients were randomly selected from a larger cohort of 70 individuals with documented multimorbidity, and four living patients were included through convenience sampling based on consent. All records were anonymized in accordance with ethical guidelines.

The data sources comprised clinical notes, referrals, discharge summaries, medication lists, records of follow-up across primary and specialist care, and entries documenting contact with family members, social services, or law enforcement. Records were excluded if they contained insufficient narrative content, pertained to isolated single-episode care (as they did not form part of a cross-sectoral or clinical pathway), or described rare or gender-specific conditions that could compromise anonymity. Non-informative administrative entries (e.g., pure scheduling or cancellations) and documents without identifiable authorship were also excluded.

Analysis began with an iterative reading to build a broad understanding of content and context, followed by an extraction of relevant narrative entries. These were categorized into clinical pathway events, focusing on points where information logistics broke down, such as duplication, delays, heterogeneous documentation, and limited availability of the patient’s voice. This process revealed recurring failure patterns across the full patient cohort, justifying the abstraction of a representative 19-year user scenario that captures the typical characteristics of information fragmentation observed in the broader dataset. The scenario was validated by a participating clinician to ensure its representativeness and fidelity to the empirical material.

Stakeholders were identified [49] and analyzed through an actor‑linkage matrix [50], highlighting uneven information flows and role dependencies. A root‑cause analysis further clarified the underlying drivers of fragmented awareness and access, including distributed responsibilities and variation in documentation routines [15]. These activities collectively delineated the high-level problem the artifact must address within the Norwegian healthcare context, where decentralized primary care and centralized specialist services must coordinate patient-centric pathways [20].

### DSR Rigor Cycle

The Rigor Cycle connects the design process to the scientific knowledge base, ensuring that the artifact is grounded in established theory and prior research [47]. The authors’ scoping review provided a structured overview of prior approaches to cross‑organizational EHR access and revealed that many existing solutions were ad hoc or technology‑driven and did not support pathway‑oriented access without duplicating records [26]. The review identified HIQ, which includes accuracy, currency, consistency, and completeness [27], as a key enabler of successful access approaches, and highlighted trust and poor information quality as major barriers. The architecture framework also draws on socio-technical theory, which helped explain why information fragmentation persists despite technical interoperability efforts. Prior work shows how heterogeneous routines and local documentation practices shape the use, interpretation, and reliability of information in clinical environments [15–17, 22, 35]. These perspectives informed the design to account for governance, provenance, context‑awareness, and workflow‑embedded access to accompany inter-organizational information logistics and coordination. National and international integration efforts further contextualized design choices, emphasizing the need for solutions that operate alongside patient‑facing services and existing infrastructures [29, 30, 33, 34]. The development of non-functional requirements and the vertical pillars was supported by established research in IG, DM, and HIQ, which emphasizes the importance of provenance, accountability, and transparency [41, 42], and aligned with legal mandates for auditability and consent handling [51–53]. The architectural design was also informed by systems‑ and software‑engineering principles, including modularity, layered decomposition, interfaces, and platform framing [36–40].

These theoretical and empirical sources collectively ensured that the artifact’s design choices were grounded in a coherent knowledge base.

### DSR Design Cycle

The Design Cycle translated the problem and the knowledge base into a prescriptive Meta‑artifact through the following design activities:

*Requirements engineering*: Based on the insights from the Relevance and Rigor Cycles, we specified high-level non‑functional environmental (Table 4) and structural requirements (e.g., governance, modularity, layered pattern, standards‑neutral interfaces) (Table 5). The non‑functional requirements defined the architectural boundaries and structures to support secure, trustworthy, and flexible inter‑organizational information logistics. These requirements prescribe alignment with existing infrastructures [29, 30, 33, 34], legal mandates and regulations for handling patient records [51–53], IG/DM prerequisites [41, 42], structural modularity and layering [36–40], and enforcement of HIQ [27].

The functional requirements (R1-R7), listed in Table 6, describe the behaviors the system must support, and were grouped into Security/Privacy (R1-R3), Awareness/Accessibility (R4-R5), and HIQ (R6-R7). Although R6–R7 are non‑functional quality attributes of the information provided, whereas R1–R5 are functional requirements (e.g., consent verification, audit, notifications, governed virtual access), they are grouped with the functional requirements because HIQ operates as a cross‑cutting mechanism governing all inter‑organizational awareness and access.

*Conceptual artifact construction*: Based on the non-functional requirements, a conceptual information-centric architecture was synthesized using established principles of modularity, interfaces, and layered decomposition [36–40]. The architectural form reflects the constraints defined by Security & Privacy, and IG/DM, and provides the structural environment in which the functional requirements (R1–R7) can be operationalized. The detailed architectural layers, components, and their interactions are presented in the Results section (Fig. 6).

### Demonstration

A hospitalization–discharge use case was selected by the Valkyrie project team to demonstrate how the architecture is intended to function during a high-risk cross-organizational transition. This scenario was chosen because empirical analyses revealed that information coordination is particularly vulnerable during such transitions, a finding also documented in the literature [54, 55]. The use case was iteratively developed by the project members and formalized into success scenarios (Table 7) and a corresponding visualization of inter-organizational interactions (Fig. 7). As a demonstration activity within the Design Cycle, its purpose was to illustrate the intended sequencing of consent checks, audit logging, awareness notifications, and governed virtual access in accordance with the specified requirements.

### Evaluation

Following Sonnenberg and vom Brocke’s framework [56], evaluation proceeded through two early‑stage, ex‑ante activities appropriate for conceptual artifacts. EVAL 1 assessed the relevance and justification of the problem using the synthesized empirical analyses, stakeholder mapping, and root‑cause analysis. This assessment established that limited awareness and restricted access to distributed records represent persistent challenges warranting an architectural response. EVAL 2 assessed the architecture’s conceptual coherence. Consistent with Hevner et al.’s descriptive evaluation methods [47], this step used scenario‑based walkthroughs (the hospitalization–discharge use case) and theory‑based reasoning grounded in socio‑technical literature [15–17, 22, 35], findings from the scoping review [26], IG/DM principles [41, 42], audit and consent regulations [51–53], and systems‑engineering concepts [36–40]. This evaluation assessed the architecture’s feasibility and consistency against the identified requirements but did not include empirical system testing, which is reserved for future prototyping.

## Results

This section contains the problem definition, user scenario, requirements, the use case outputs, and the evaluation findings.

### Problem definition

The problem definition starts with the user scenario, followed by stakeholder identification, information-flow intensity, root causes, and the problem definition.

#### User scenario – parallel clinical pathways and convergence

**Fig. 2:**
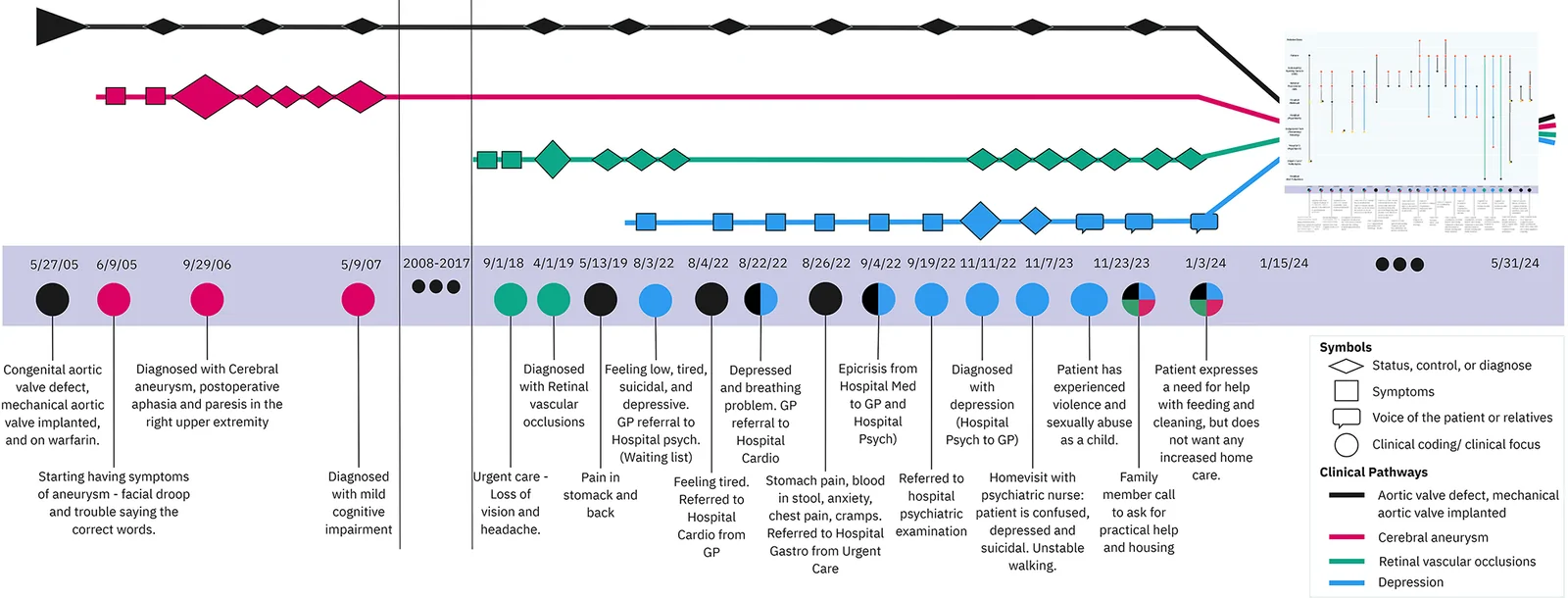
Visualization of healthcare events, actions, and the information flow between actors along clinical pathways for a patient with mental and physical health issues

**Fig. 3:**
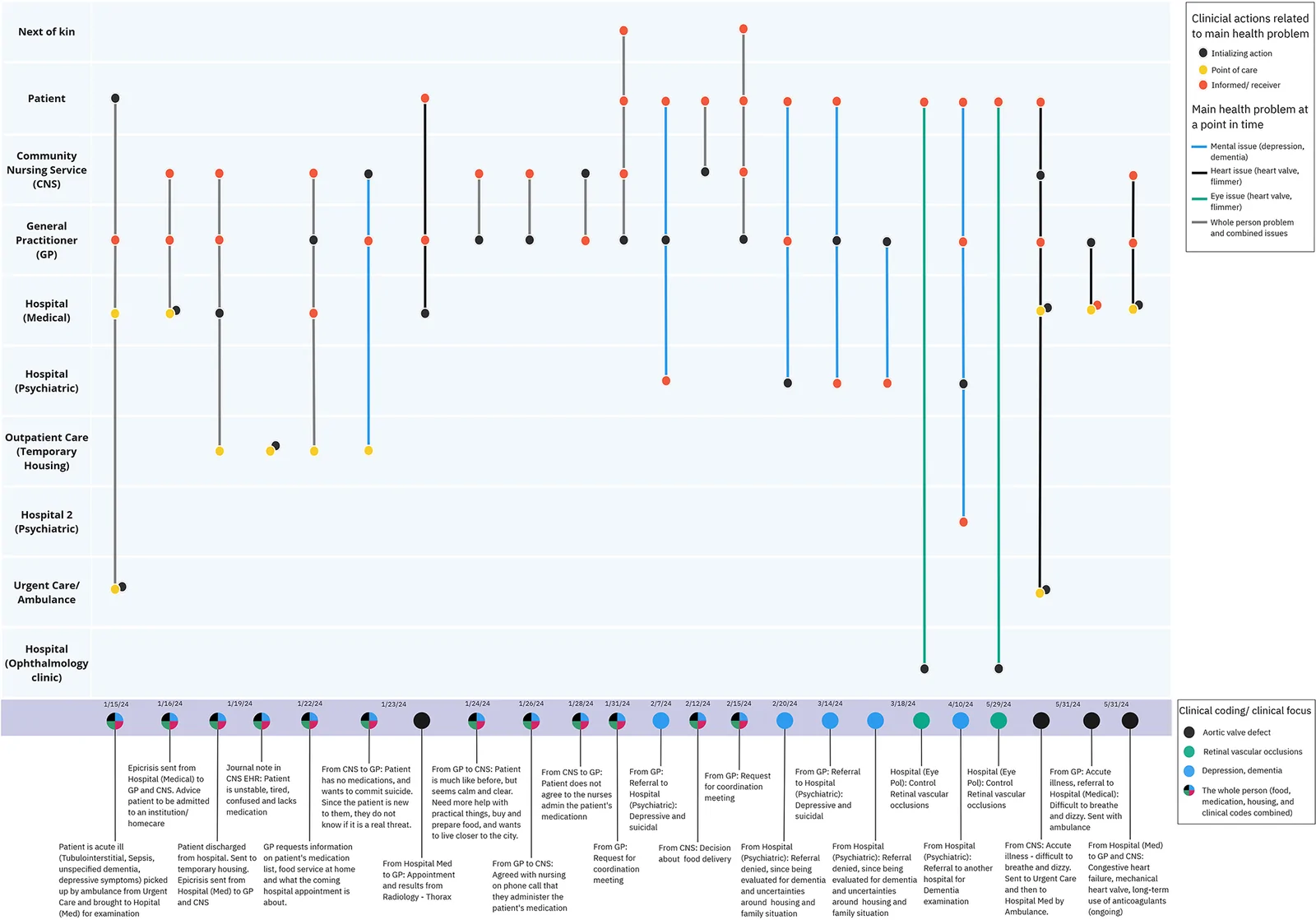
Visualization of healthcare events, actions, and the information flow between actors for a patient with mental and physical health issues when the clinical pathways converge

**Fig. 4:**
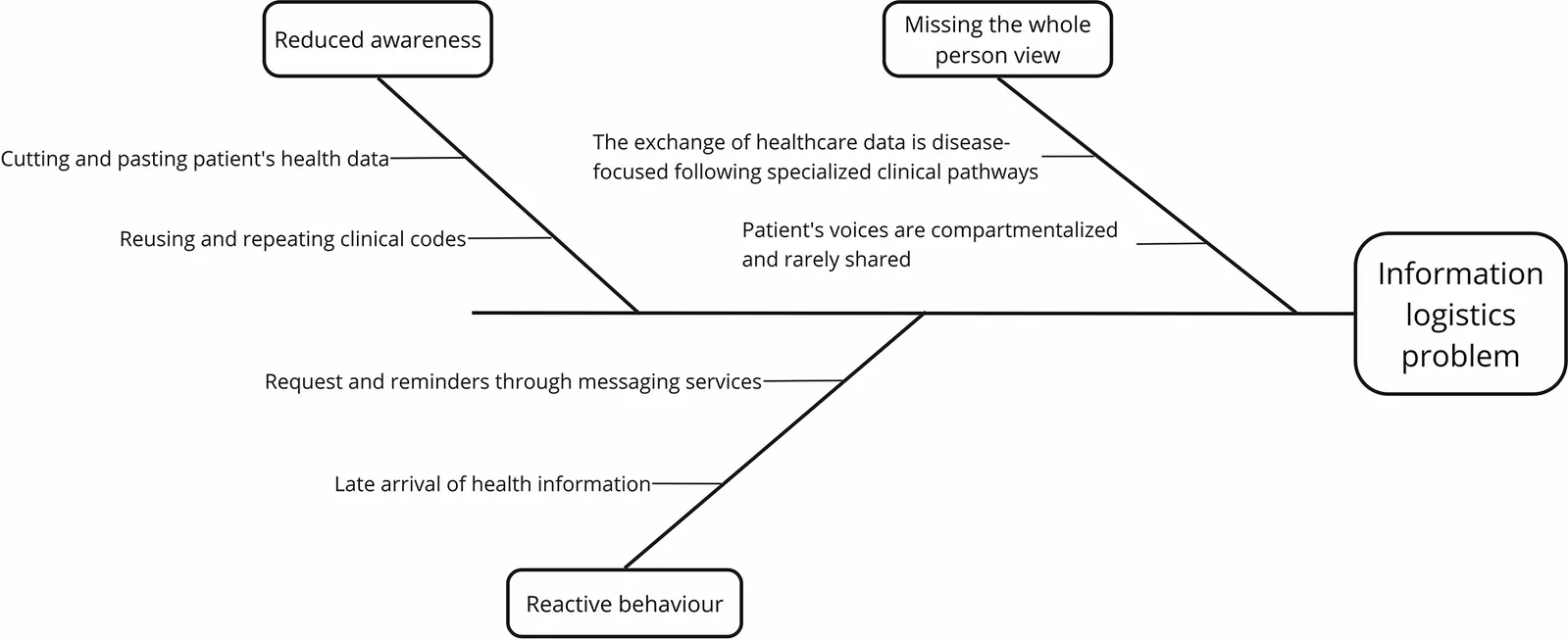
Root-cause analysis of the information logistics problem

The longitudinal user scenario tracks a multimorbid patient’s 19-year clinical pathways (2005–2024), demonstrating interactions across specialist and primary care providers (Fig. 2). The full chronology of events and actions is detailed in Supplemental Digital Appendix A. Initially, the patient managed distinct parallel clinical pathways (Cardiovascular, Neurological, Vascular, and Mental Health) until 2022. This complexity intensified significantly at the beginning of 2024, exposing systemic failures in information logistics during an acute care transition and subsequent discharge (Fig. 3). This critical convergence of pathways confirmed systematic findings of data duplication (F1, F2), delays in timely information logistics (F4), and the compartmentalization of essential patient input (F6), as detailed in Table 1. The root-cause analysis (Fig. 4) reveals that this information fragmentation creates uncertainty in care decisions, risks patient safety, and necessitates time-consuming manual work for clinicians.

Table 1 lists the identified problems, confirming systematic challenges in inter-organizational DM. These include frequent data duplication through cutting and pasting (F1) and code reuse (F2), significant delays in timely information logistics (F4), the lack of inter-organizational access (F5), and the compartmentalization of essential patient input (F6), all of which compromise HIQ.

**Table 1:** Identified problems, assumed causes, problem groups, and possible consequences elicited from the user scenario

#	Identified problems	Assumed causes	Problem groups	Possible consequences of the problems
F1	Cutting and pasting patient data to achieve an overview of the patient’s illness	Missing patient summary	Data duplication	Lowered HIQ Added manual work Uncertainties in clinical decision making
F2	Reusing and repeating clinical codes for each new contact	Clinical codes are used for different purposes (documentation and reimbursement)	Data duplication	Lowered HIQ Added manual work Uncertainties as to whether the codes relate to a pathway or a particular episode.
F3	Sending messages with reminders and requests for information	Missing access to patient records and medication lists	Unintended use (Appropriation)	Added manual work Need to await responses Uncertainties as to whether all relevant clinicians receive messages.
F4	Health information, treatment plans, and medication lists arrive late at the point of care	The clinical person responsible is also responsible of informing others.	Untimely information logistics	Added manual work Need to await responses Uncertainties in clinical decision-making Patients as information carriers
F5	Symptoms can indicate both physical and mental health conditions but are not easily shared across clinical domains	Missing access to essential information	Missing integrated, patient-centric pathway view	Can increase redundant work and limit effective examination, diagnoses, and treatment
F6	Patients’ individual needs are mostly compartmentalized, and not easily shared	There is no common recording and sharing of patient’s voices inter-organizationally.	Missing integrated, patient-centric pathway view	Missing patients’ voices can affect the treatment quality and progression.

Table 2 lists the identified stakeholders followed by a description of the stakeholders.

**Table 2:** Description of the stakeholders identified during the construction of the user scenario

Identified stakeholders	Description of stakeholders
Patient	A person who, due to symptoms or illness, becomes a patient and enrolls into the healthcare system. Although categorized as a patient by healthcare and belonging to one or more patient groups, their personal preferences and needs may result in different behaviors to identical treatment and medication options.
Next of kin	It is the patient’s closest relation, appointed by the patient, and is often a family member. Next of kin has the role of guardian for the patient and may be both informed and participate in decision making concerning the patient. They have broader privileges than relatives, including access to patient records.
General Practitioner (GP)	Healthcare professionals who specialize in general medicine. GPs are often the first contact point for citizens with symptoms of illness and encounter less severe conditions than specialists at a hospital and can prevent problems from escalating. GPs refer patients to specialists and receive discharge reports (epicrisis) from specialists.
Medical physician	Specialist doctor at the hospital medical department, examining and treating patients with physical (somatic) conditions.
Psychiatrist	Specialist doctor working in the psychiatry department at the hospital, diagnosing and treating patients with mental health conditions, such as depression, anxiety, and bipolar disorder. Psychiatrist 2 identified through the user scenario was a specialist in adult psychiatry, especially related to diagnosing dementia.
District nurses/ home care nurses	Nurses or nursing assistants employed by the municipalities and work in care units for patients at home or at care institutions for shorter or longer stays.
Urgent care (UC)	Organized under the municipalities offering acute medical services after GP’s closing time. Like the GP, they can refer patients to the hospital.

As clinical pathways converged (Fig. 3), more stakeholders became involved, interactions increased, and the information flow intensified. Using these insights, an actor-linkage matrix (Table 3) was constructed, showing the GP as the central hub with the highest intensity of information flow, particularly with CNS, and the medical physician. While CNS initiated the flow with the GP, the GP acted as the primary gatekeeper to specialized care. The patient exhibited minimal information flow, primarily as a receiver, reflecting the compartmentalization of the patient’s voice across organizations. In Table 3, blank cells indicate no information flow, while cells with question marks suggest that information flow is likely occurring but is not documented. The stars indicate the occurrence of information flow between stakeholders, with the number of stars representing the information flow intensity.

**Table 3:**
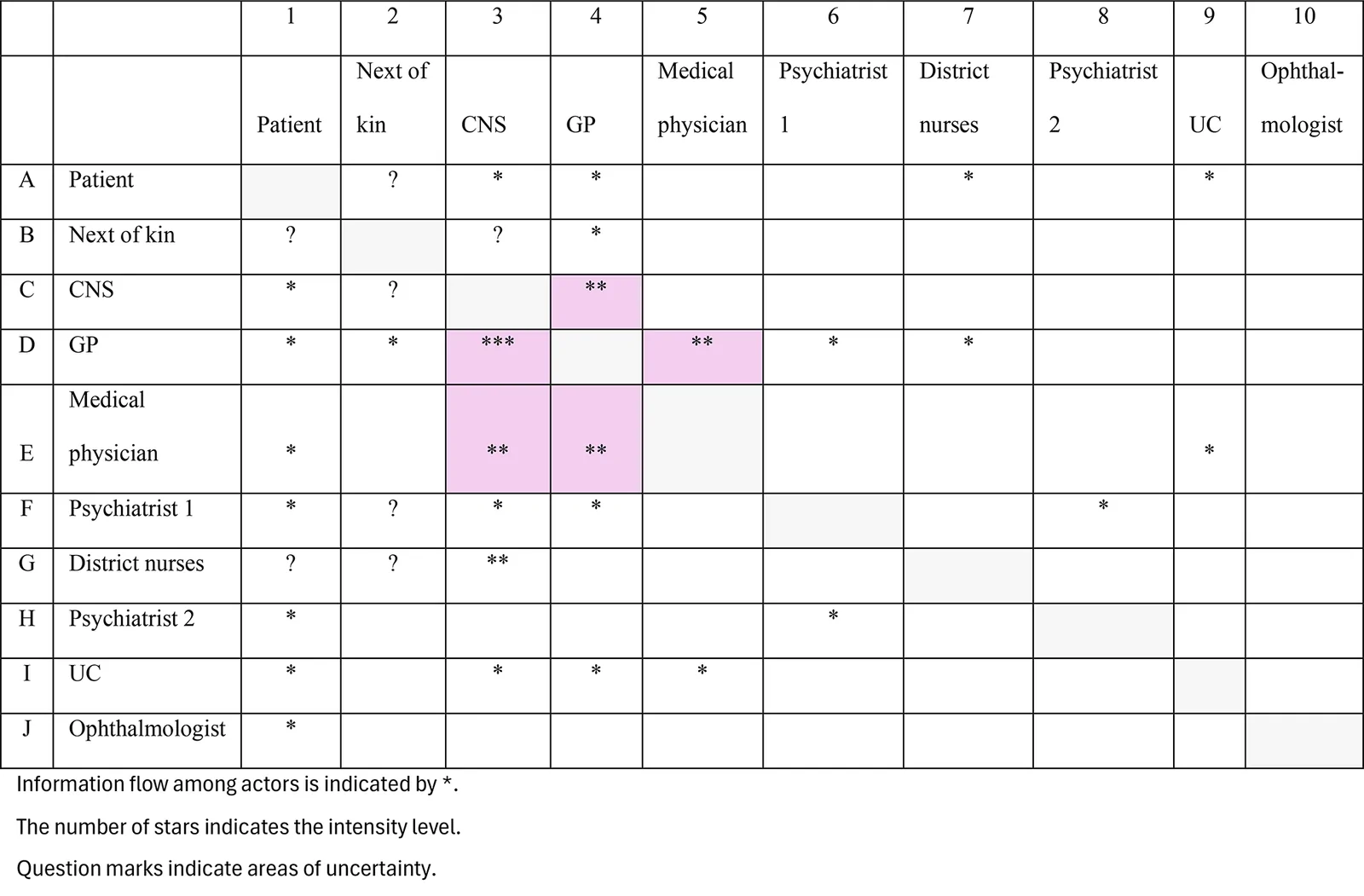
Mapping the intensity of the information flow between the identified stakeholders using an Actor-linkage matrix

Using the synthesized empirical findings (Table 1) and the derived root-cause analysis (Fig. 4), we assert that information fragmentation systematically creates uncertainty in care decisions, elevates patient safety risks, and necessitates substantial time-consuming manual clinician effort to assemble data from multiple sources. The analysis confirms that the information logistics crisis stems fundamentally from two persistent, pervasive system deficiencies: (1) a lack of awareness regarding parallel treatment activities elsewhere, and (2) limited virtual access to distributed patient records. This situation highlights a growing and urgent demand for comprehensive and timely information logistics when complex clinical pathways converge. Accordingly, the problem is defined as:

*Current patient and clinical integrations suffer from inter-organizational information logistics*,* which limit awareness of patients’ voices and treatment activities at other providers*,* and reduce access to distributed patient records. This restricts clinicians’ proactive use of essential health information at the point of care and constrains patients’ ability to share their voices and participate in care decisions.*

The synthesis of longitudinal patient data with inter-organizational stakeholder mapping confirms that information fragmentation is not merely a technical byproduct but a systemic failure of current health information logistics. The root-cause analysis (Fig. 4) identifies a pervasive ‘lack of awareness’ and ‘lack of access’ as the primary drivers of clinical uncertainty and manual workload (F1–F6). However, this empirical problem definition also carries a crucial architectural mandate: opening patient records to resolve these gaps inherently introduces socio-technical side effects, specifically the risk of accidental disclosures of sensitive information and the exposure of discrepancies in work routines and documentation practices across organizations. These factors heighten concerns regarding DM, HIQ, security, and privacy. Consequently, successful mitigation requires that the prescriptive architecture framework explicitly operationalize governance and management factors as non-functional constraints, providing the structural foundation necessary to sustain inter-organizational trust and collaboration.

### Requirements and framework

Building on the empirical problem defined in the previous section, we now transition to the systematic definition of requirements necessary to address fragmentation and support robust information logistics. As posited within the DSR paradigm, the solution artifact, the conceptual information architecture framework, must function as a prescriptive design knowledge (Meta-artifact), rigorously guiding the translation of problem constraints derived from the Relevance Cycle into requirement specifications.

### Conceptual framework outline and governing pillars

**Fig. 5:**
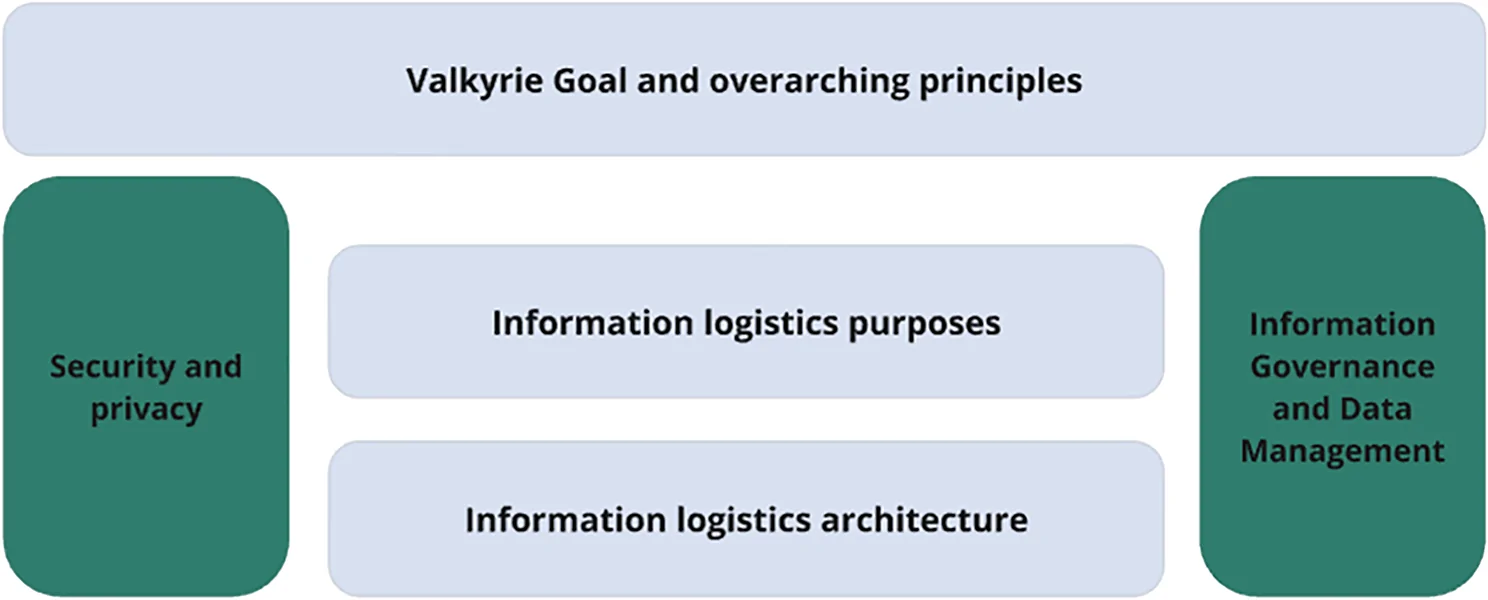
Outline of the conceptual information architecture framework

To guide this translation, we outline the conceptual information architecture framework (Fig. 5), synthesizing core insights from the preceding scoping review and the foundational Valkyrie principles. This conceptual model is structured to integrate the overarching Valkyrie goals with authoritative, vertical pillars. Crucially, addressing the lack of inter-organizational awareness and access inherently heightens concerns about security, privacy, and HIQ, risking the formation of tension and distrust among collaborating stakeholders. Therefore, the framework explicitly prescribes the necessity of Security and Privacy, and IG/DM as non-functional constraints that must be aligned with system-wide standardization initiatives to enable inter-organizational trust and collaboration.

### Environmental requirements

Building upon the abstract conceptual information architecture framework outlined (Fig. 5), we now systematically specify the high-level non-functional requirements that translate the defined problem of information fragmentation into tangible architectural constraints. These requirements dictate the essential contextual boundaries (environmental) constraints and the internal organization (structural) required to govern and realize the solution. Non-functional requirements, being generic and widely applicable, ensure fundamental qualities such as usability, security, and sustained interoperability [43].

**Table 4:** Description and justification of the inter-organizational high-level non-functional environmental requirements

Inter-organizational high-level non-functional environmental requirements	Description and justification
The system should adhere to Valkyrie’s goal and overarching principles.	It is a prerequisite that the artifact is aligned with Valkyrie’s goals and principles, facilitating integrated, patient-centric information logistics [3].
The system should be flexible to adapt to external regulations, standards, and recommendations.	Due to the dynamic interplay in healthcare [15, 17, 18] and the differences in funding, priorities, and autonomy among healthcare providers, changes will likely occur across various time scales, requiring a flexible and adaptable system.
The system should handle conflicts of interest and HIQ issues through inter-organizational IG.	IG can improve normative and functional integration, reducing tension and handling conflicts of interest [28], which can arise when closed systems are opened to outsiders and differences in processes, procedures, and documentation routines are revealed [26].
The system should incorporate existing national infrastructures and components.	It is crucial for the system’s overall success and its attractiveness to other healthcare domains and providers that national infrastructure and components are incorporated [14].
The system should ensure security and privacy by design.	Ensuring that health information is safe and secure is crucial for patients’ and clinicians’ trust in the healthcare system and each other, and ultimately, the treatment quality [26].

The environmental requirements define the broader contextual constraints, derived from the problem domain and the overall Valkyrie objectives (Fig. 5). These requirements (Table 4) prescribe adherence to existing national infrastructure and external regulations, and socio-technical conditions, mandating that the system must proactively handle conflicts of interest and pervasive HIQ issues through rigorous inter-organizational IG, which is crucial when opening traditionally closed systems.

### The prescriptive information logistics architecture

**Fig. 6:**
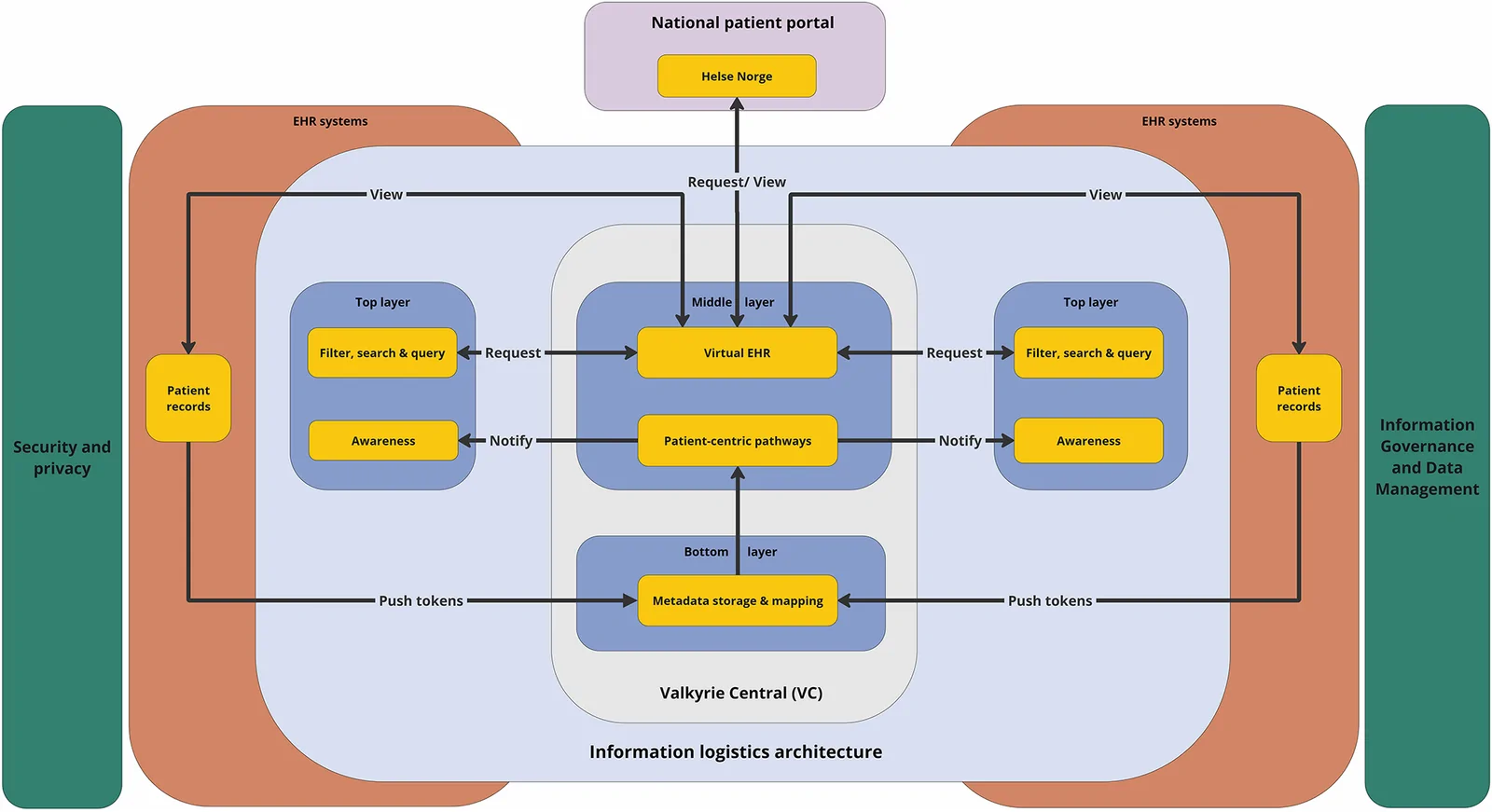
The conceptual information logistics architecture with layers and modules interacting across two EHRs, a national patient portal, and the Valkyrie Central (VC)

While the environmental requirements (Table 4) define the external constraints, the detailed conceptual architecture explicitly describes the internal composition and structures required to solve the core problem. Figure 6 presents the conceptual information logistics architecture, visualizing the rigorous, layered, and modular structure prescribed to operationalize the framework and address the root causes and problem definition identified (lack of awareness and lack of access).

The architecture is systematically divided into three horizontal layers, constrained vertically by the authoritative Security and Privacy and IG/DM pillars:

#### Top Layer (Clinician/Patient Interaction):

This layer directly addresses fragmented access and limited awareness by embedding user interfaces within existing EHRs and the National Patient Portal, thereby reducing workload by eliminating the need for additional logins. It introduces two key modules that respond directly to the defined problem:

- Awareness Module: This module supports clinicians with contextual notifications of relevant activities at other providers regarding a patient’s concurrent treatment activities. These notifications are enabled by structured, lightweight information tokens (metadata, clinical codes, location pointers) pushed from the underlying EHR systems and organized coherently into a patient-centric pathway in the middle layer, mitigating the “lack of awareness” problem.
- Filter, Search, and Query Module: This module enables clinicians to actively filter, search, and query the information tokens from the distributed patient records. This module relies on the Virtual EHR located in the middle layer to provide read-only views of distributed patient records, thereby mitigating the “lack of access” problem.

#### Middle Layer (Pathways and Virtual Access):

This layer handles notifications and requests and contains mechanisms for translation and context-awareness to order information tokens into relevant patient-centric models. It includes the Patient-centric pathway module, which maps information tokens into coherent pathway models to support coordination and awareness, and the Virtual EHR, providing the on-demand, read-only views of the patient’s distributed records necessary for detailed clinical review. Importantly, these modules must work contextually together to ensure smooth transitions between the pathway overview and the detailed clinical review, supporting different clinical reasoning processes.

#### Bottom Layer (Secure Storage and Provenance):

This layer ensures that the tokens are securely stored and compliant with legal requirements [51], guaranteeing integrity and traceability. This foundational control is vital for reinforcing the IG/DM pillar and sustaining interprofessional trust.

Together, this strategic layering, separating concerns across user interaction, pathway logic, and secure storage of information tokens, facilitates decoupling information from application constraints. This decoupling approach is essential for achieving the necessary long-term DM flexibility, enabling future information organization and reorganization independently from underlying application data models [2, 21, 23].

### Structural requirements

The internal organization and design decisions visualized in the conceptual architecture (Fig. 6) are formally specified by the structural requirements. These requirements serve as prescriptive constraints to manage complexity and ensure the artifact functions as reusable design knowledge. As formalized in Table 5, the structural requirements are defined to maximize long-term utility:

- Modularity and Layered Pattern: The requirement for a modular design and layered pattern approach (Table 5) is explicitly mandated to promote the creation of reusable components and ensure ease of system maintenance and flexibility when incorporating internal or external additions.
- Standards-Neutrality: The prescription to avoid vendor lock-in by supporting open standards and APIs (Table 5) ensures DM flexibility and long-term architectural stability, allowing the framework to support international health information standards (e.g., HL7 FHIR, openEHR) without imposing a single rigid approach.

**Table 5:** Description and justification of the inter-organizational high-level non-functional structural requirements

Inter-organizational high-level structural Requirements	Description and justification
The system architecture should have a modular design and a layered pattern approach.	A modular architecture promotes reusable components and can also make the architecture flexible to incorporate new internal or external additions to benefit the healthcare system’s stakeholders. Modularity and layered architectures promote the separation of concerns and ease system maintenance [36, 37, 40].
The system should avoid vendor lock-in by supporting open standards and APIs.	Open standards and APIs can help lower entry barriers and empower healthcare providers to change modules or systems without data loss or significant conversion costs [39, 57].
The system should be embedded into local patient record systems/ clinical workflow.	By following modularity [38] and virtualization ideas, the system’s user interface should be embedded into existing applications and workflows, which can help reduce the number of user interfaces and logins, and avoid vendor lock-in [57].
The system should protect health information from unauthorized use.	The information tokens representing patients’ pathways must be securely stored and be available to authorized clinicians only. This is regulated by the Patient Journal Law § 22 [51], and is essential for building trust among stakeholders.
The system should offer high availability and accessibility.	As a general requirement, the system should provide availability and accessibility that is equal to or greater than that of the distributed patient record systems it depends on, which also affects the system’s load time [26].
The system should support stakeholders in navigating and exploring patient pathway information according to personal preferences.	Because of the dynamic and contextual nature of healthcare work [18, 22], clinicians must be offered various methods of navigating the information provided such as filtering, searching, and querying the information, with limited predefined navigation patterns.

These specified structural requirements confirm the prescriptive nature of the conceptual architecture, rigorously detailing the internal conditions necessary for the artifact to govern information assets and enable inter-organizational information logistics.

### Functional requirements

Having formalized the non-functional constraints that govern the architecture’s environment and internal structure, we now proceed to specify the functional requirements. Functional requirements detail how the envisioned system must interact with its users to rigorously address the identified information fragmentation problem [43]. These requirements translate the vertical pillars and components into tangible system behaviors.

The functional requirements (Table 6) are systematically grouped into three core categories reflecting the authoritative vertical pillars of the conceptual framework and the defined problem: Security and Privacy, Awareness and Accessibility, and HIQ.

#### Security and privacy (R1–R3)

The Security and Privacy requirements ensure the technical solution adheres to the legal and professional standards necessary to sustain stakeholder trust when accessing distributed patient records. They mandate transparent audit trails (R1), aligned with laws requiring logging of all clinical access to confidential information [51]. Furthermore, the system must control informed consent (R2), acknowledging the patient’s right to limit access to their confidential information [52, 53]. Operational efficiency is maintained by requiring the reuse of existing organizational authentication and authorization mechanisms (R3) wherever possible, avoiding the introduction of new logins or maintenance overhead [26].

#### Awareness and accessibility (R4–R5)

These requirements directly operationalize the fundamental issues identified in the root-cause analysis and the problem definition: lack of awareness and lack of access. The system must deliver notifications of parallel treatment activities (R4) to ensure clinicians gain timely awareness of concurrent events and medications. When further details are needed, the system must provide virtual access to distributed patient records (R5) to address the access gap. Critically, R5 also explicitly mandates the integration of the patient’s voice and preferences, mitigating the empirically observed problem of compartmentalized patient input that affects treatment quality (F6) [12].

**Table 6:** Description and justification of the inter-organizational high-level functional requirements grouped into Security and privacy, Awareness and accessibility, and Health Information Quality

#	Inter-organizational high-level functional requirements		Description and justification
	**Security and privacy**		
R1		The system should provide access logs for transparency.	The Patient Journal Law § 22 states that all Health Information Systems containing confidential information must ensure audit trails of when and who has accessed the records, the purpose of accessing, the organization they belong to, and the time interval for accessing [51]. Thus, creating audit trails and making them transparent is required.
R2		The system should control patients’ informed consent before allowing clinicians to access patient records.	The Health Personnel Law § 25 and § 45 states that confidential information can be accessed by authorized clinicians and shared with cooperating personnel when necessary to provide responsible health care, unless the patient refuses [52]. Patients can refuse partial or complete access to their confidential information and withdraw it at any time, according to the Patient and User Rights Act § 5 − 3 [53]. Thus, it is necessary to check if patients have consented before clinicians can access all or parts of their records, which also relates to the Cost-effectiveness principle of HIQ [27].
R3		The system should reuse existing authentication and authorization mechanisms whenever possible.	Ensuring clinicians outside the organization can be trusted for access using their organizational login credentials and accessing policy is important for a successful inter-organizational information logistics architecture [26]. If possible, the system should not introduce additional logins to avoid maintenance costs on an inter-organizational level.
	**Awareness and accessibility**		
R4		The system should notify clinicians of parallel treatment activities and medications.	This requirement is elicited from the problem definition stating that multimorbid patients need integrated care, which means that clinicians must become aware of treatment activities elsewhere to ensure qualitative and safe patient treatment at the point of care.
R5		The system should provide stakeholders with virtual access to distributed patient records and patients’ voices.	This requirement is elicited from the problem definition, stating that clinicians lack inter-organizational access to distributed patient records and should be provided with virtual access. This requirement is in line with the Health Personnel Law § 25 and § 45 [52]. In patient-centered care, access to patients’ needs and wants can be important, influencing the treatment progression and quality of life [12].
	**Health Information Quality**		
R6		The system should provide accurate, current, consistent, and complete health data.	This requirement reflects the data quality aspects of HIQ with health data as valid (Accuracy), up to date (Currency), consistent (Reliability), and complete (Completeness) to make informed decisions [27].
R7		The system should provide understandable and useful information for authorized personnel when and where needed.	This requirement reflects the data context and relevance aspects of HIQ, stating that all data, whether (hand) written, transcribed, or printed, should be understandable (Readability), useful to the stakeholders (Usefulness), and protected from unauthorized use (Confidentiality) [27].

#### Clarification and role of HIQ requirements (R6–R7)

Requirements R1–R5 specify functional system behaviors, such as consent verification, auditability, notifications of concurrent treatment activities, and governed virtual access, addressing the identified gaps in awareness and access. In contrast, R6 and R7 describe non‑functional requirements concerning the quality of the information provided, including accuracy, currency, consistency, completeness, readability, and contextual usefulness. Although these are quality attributes rather than discrete functions, they are intentionally grouped with the functional requirements because HIQ is a central cross‑cutting mechanism within this architecture.

#### Operationalizing information governance and data management through HIQ (R6–R7)

R6 and R7 operationalize the framework’s vertical IG/DM pillars, ensuring that any inter-organizational access is supported by governed, trustworthy, and contextually interpretable information [26]. R6 establishes the requirement that information made available across organizations must be accurate, current, consistent, and complete [27], directly addressing failures such as duplicate patient data (F1, F2) and inconsistencies arising from fragmented documentation.

R7 addresses the contextual dimension of information use by requiring that data be readable, relevant, and useful when and where needed, contributing to reduced cognitive burden and mitigating the effects of untimely information logistics manual workarounds (F3, F4). Together, these requirements ensure that notifications and virtual access do not simply expose distributed data but provide governed, high‑quality information aligned with the demands of pathway‑oriented information logistics.

### Use case results and evaluation findings

This section reports the results of the use case, showing how the conceptual architecture operated during a cross‑organizational care transition and how the evaluation outcomes (EVAL 1 and EVAL 2) manifested during the scenario walkthrough.

### Use case results

A focused use case was developed to illustrate how the conceptual information-centric architecture operates during a high-risk transition: a patient’s hospitalization and subsequent transfer to community care. The scenario reflects typical cross-organizational information needs and exposes how the architecture functions when clinical responsibility shifts across providers.

Table 7 presents the eleven success scenarios (S1-S11). Together, they show the sequence of interactions during transition that occur when users at different organizations request access to distributed patient information, receive notifications, and obtain governed, read-only views of patient records.

The sequence begins when the medical physician requests access to the Virtual EHR to review the patient’s history (S1). The system checks the patient’s registered consent (S2) and, subject to confirmation, grants the physician read-only access to relevant GP and CNS records using existing organizational authentication (S3). All access is logged (S4). The system then notifies downstream stakeholders, the GP and CNS, of the ongoing examination and discharge preparations (S5).

As the patient approaches discharge, the GP and CNS request updated information through Virtual EHR (S6). The system again checks consent (S7) before granting access to updated hospital records (S8) and logging all actions (S9). When the patient arrives at the community institution, the system notifies the hospital physician (S10) and subsequently enables the GP and hospital physician to view CNS records via Virtual EHR (S11).

**Table 7:** Use case description and success scenarios of the future system’s inter-organizational virtual information logistics during a patient’s hospitalization and transfer to a community service institution

#	Use case description
	**Precondition**
	The patient is hospitalized after being sent from the Urgent Department to Hospital Medical in an ambulance, suffering from tubulointerstitial nephritis and sepsis. The GP and the CNS have earlier involvement with the patient and are informed of the patient’s hospitalization through a messaging service by the medical physician.
	**Main success scenario**
S1.	During the patient examination, the medical physician requests access to the Virtual EHR to review the patient’s medical history.
S2.	The system checks the patient’s informed consent with the National Patient Portal.
S3.	The system grants the medical physician access to patient records at the GP and CNS using local organizational credentials.
S4.	The system logs the medical physician’s search activity.
S5.	The system notifies the GP and CNS of the patient’s hospital examination and discharge preparations.
S6.	The GP and CNS request updates on the patient’s medical and medication status through Virtual EHR before hospital discharge.
S7.	The system repeats the informed consent check with the National Patient Portal, this time for the GP and CNS.
S8.	The system grants the GP and responsible nurses at CNS access to view the patient’s medical status and medication list from the hospital’s patient records using local organizational credentials.
S9.	The system logs the GP and CNS search activities.
S10.	The system notifies the medical physician about the patient’s arrival at the community institution.
S11.	The system provides the GP and medical physician with access to CNS’s patient records via Virtual EHR.

**Fig. 7:**
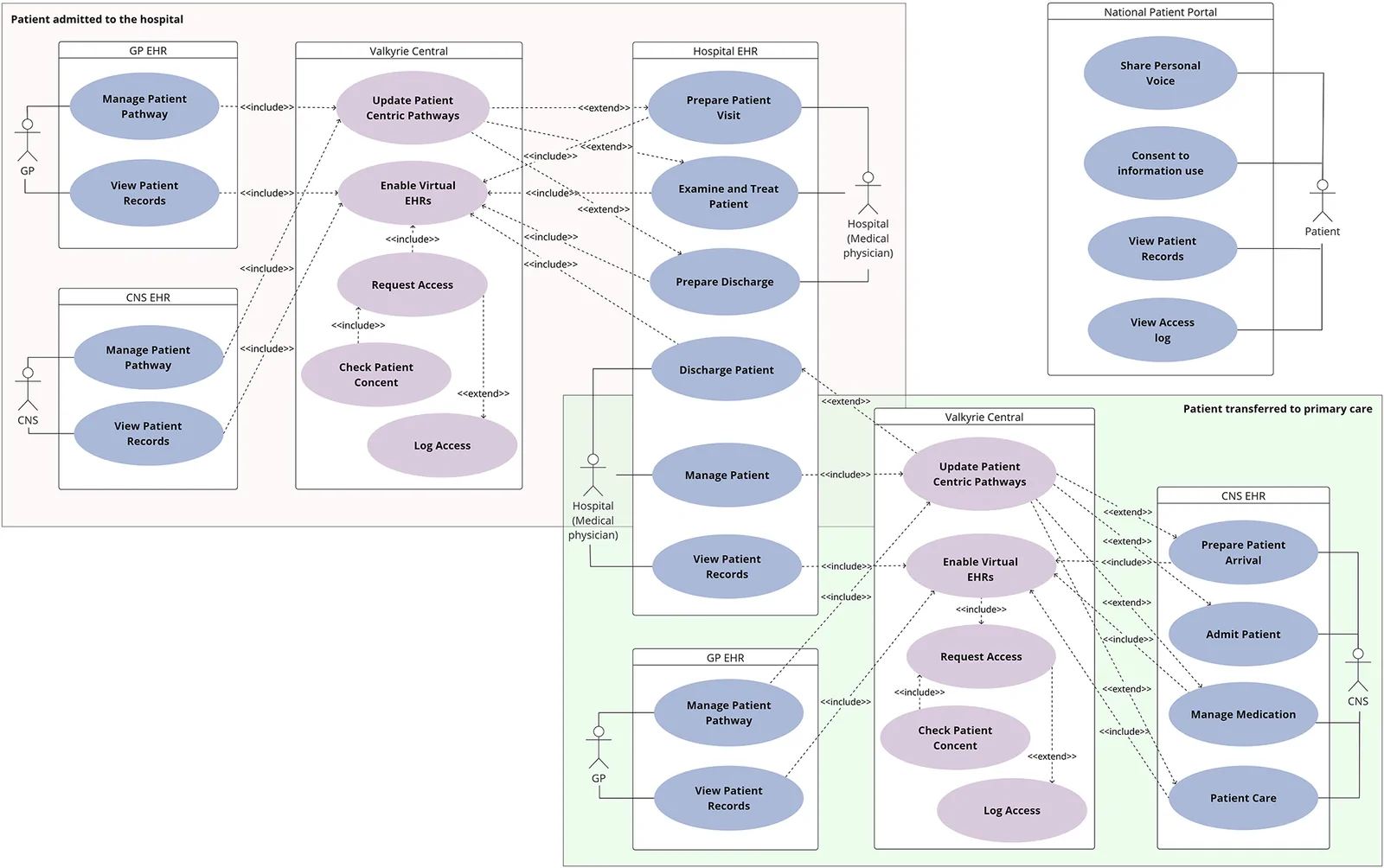
Use case of the inter-organizational information logistics based on the functional requirements

Figure 7 visualizes these inter-organizational interactions, illustrating how information flows through the coordinating component Valkyrie Central (VC). VC handles notifications, consent checks, access decisions, and the retrieval of read-only, source-controlled views from distributed EHRs without replicating records.

Across S1–S11, the use case output demonstrates how the prescribed functions, notifications (R4), governed virtual access (R5), consent-controlled disclosure (R2), transparent audit logging (R1), reuse of organizational credentials (R3), and provision of current read-only data (R6–R7), materialize within clinical transition. The use case shows how the architectural components work together to support pathway-oriented information flows and reduce reliance on manual workarounds, such as phone calls and unstructured messaging.

### Evaluation results

The evaluation activities examined whether the identified problem was sufficiently substantiated (EVAL 1) and whether the proposed architecture aligns with the requirements and constraints derived earlier (EVAL 2).

#### EVAL 1 – Problem justification (Results)

EVAL 1 found that the synthesized empirical material, comprising the 14 multimorbid patient trajectories, the representative 19-year scenario, stakeholder mapping, and the root-cause analysis, consistently reflected the two systemic deficiencies defined in the problem formulation: lack of awareness of parallel treatment activities and lack of access to distributed patient records. These patterns were recurrent across care levels and organizational contexts and manifested in duplicated entries, delayed availability of essential information, and limited visibility of patients’ voices. The use case walkthrough reproduced these conditions, showing where awareness and access gaps typically arise during patient transition across organizations.

#### EVAL 2 – Design consistency (Results)

EVAL 2 assessed the consistency of the architecture with the requirements and design constraints. The walkthrough of the use case revealed that the architecture executed the expected sequences of consent checks, audit logging, notifications, and retrieval of governed, read-only data in accordance with R1–R7. During S1–S11, the system applied the vertical pillars (Security & Privacy, IG/DM) as prescribed, ensuring that access was logged, consent was consulted, and current information was retrieved directly from source systems. The layering of interaction, pathway logic, and secure storage behaved as intended, separating user-level actions from provenance and storage operations. Taken together, these outcomes indicate that the components behaved in a manner consistent with the structural and functional requirements defined earlier.

## Discussion

This study proposes a conceptual information-centric architecture framework for strengthening inter‑organizational information logistics in integrated care. Guided by the DSR paradigm, the work progressed from an empirically grounded problem definition through high‑level requirements to a prescriptive architectural solution, followed by a scenario‑based demonstration and a descriptive evaluation. The Discussion interprets these findings, situates the framework within relevant theory and related work, and reflects on its contributions, implications, and limitations.

### Summary of the DSR cycles

This study followed Hevner’s three‑cycle Design Science Research framework to ensure methodological rigor and relevance. The Relevance Cycle synthesized empirical insights from 14 multimorbid patient trajectories and a representative 19‑year scenario, supported by stakeholder mapping and root‑cause analysis. Together, these revealed systemic failures in awareness, access, duplication, and untimely logistics. EVAL 1 showed that these deficiencies were consistently observed across cases, confirming the substantive and recurrent nature of the problem that the artifact must address. The Rigor Cycle incorporated knowledge from the authors’ scoping review of inter‑organizational EHR approaches, socio‑technical theory, literature on IG, DM, and HIQ, and systems‑ and software‑engineering foundations, aligned with legal mandates for auditability and consent handling. These sources informed the architectural requirements, constraints, and quality mechanisms embedded in the framework. The Design Cycle translated these insights into a layered, modular architecture and demonstrated feasibility‑in‑principle through a hospitalization and discharge use case, illustrating how the architecture functions across organizational boundaries. Collectively, the three cycles ensured that the Meta‑artifact is both theoretically grounded and empirically motivated while remaining open to future instantiation and testing.

### Socio‑technical foundations and implications

Inter‑organizational information fragmentation emerges not merely from technical limitations but from socio‑technical dynamics embedded in clinical work. Variation in documentation routines, differences in professional responsibility, and the contextual nature of coordination all influence how information is produced, interpreted, and shared. EVAL 1 reinforced this view by showing that awareness and access gaps manifested at the intersections of organizational boundaries, where socio‑technical variability is greatest. These findings underscore the need for architectures that not only facilitate data exchange but also embed governance structures, provenance, and quality controls to address the social and organizational contingencies shaping information use. The framework’s emphasis on contextualization, workflow alignment, and the integration of the patient’s voice reflects these socio‑technical realities.

### Engineering foundations and architectural implications

The architectural structure draws on established systems and software‑engineering principles, including modularity, layered decomposition, and the reduction of interdependencies through interfaces. These principles support separation of concerns, manage complexity, and enable components to evolve independently. Through EVAL 2, the scenario walkthrough showed that this layered structuring behaved as intended: user‑level interactions triggered pathway‑level interpretation and governed retrieval from the secure storage layer without exposing underlying data models. These results indicate that the architecture’s layered and modular structure operates as intended, supporting information-centric coordination in distributed environments.

### From application-centric to information-centric architecture

This study also contributes to the broader shift from application-centric to information-centric architectural thinking. Existing approaches identified in the scoping review often depend on physical record replication or assume homogeneous data models [26]. These strategies struggle with synchronization burdens, versioning issues, and heterogeneous documentation practices. The information-centric architecture presented here addresses these limitations by decoupling information from application boundaries and reorganizing patient-relevant data into information tokens. By separating user interaction, pathway logic, and secure storage, the framework provides a structured “TO BE” vision that supports long-term flexibility in data management. This architectural direction supports adaptability and provides a foundation for future prototyping and research in data-driven initiatives such as process mining [58] and learning health systems [59, 60]. It may also serve as a basis for exploratory work on AI-enhanced filtering, summarization, and reasoning [61], while maintaining organizational autonomy and data sovereignty.

### Advancing inter-organizational information logistics

The use case (Fig. 7) illustrates how the architecture’s prescribed mechanisms work together to address the systemic gaps identified in the problem definition. By enforcing consent checks and transparent audit logging before granting access to distributed records, the system embeds accountability and trust directly into each information-sharing event. These governance constraints ensure that access to clinical details is consistently controlled, visible, and compliant with legal requirements, thereby supporting trust between organizations. The use case also shows how timely notifications can reduce reliance on manual information-chasing and unstructured messaging by alerting downstream clinicians to key events such as examinations, discharge preparations, and transfers. By ensuring that clinicians receive current, source-controlled information through read-only virtual access rather than replicated records, the architecture helps mitigate data duplication, outdated information, and documentation inconsistencies. Furthermore, by making the patient’s voice and preferences visible across organizations (R5), the framework supports more informed planning and draw attention to social factors, such as housing and financial concerns, that may influence referral decisions and treatment progression. Taken together, these mechanisms illustrate how the framework can support coordinated transitions, reduce information delays, and provide high-quality, context-relevant patient information during inter-organizational care processes.

### Privacy and access balance

Opening access to distributed patient information across organizations introduces important privacy considerations. The framework mitigates these risks by sharing lightweight information tokens instead of full records, verifying patient consent before disclosing details, and enforcing transparent audit trails for every access event [51–53]. These controls help address concerns about unintended disclosures, discrepancies in documentation practices, and variability in local governance routines. The architecture also restricts information exposure to what is relevant for the current task, reducing unnecessary visibility of sensitive information. Together, these mechanisms support a balanced approach that upholds transparency and safety without undermining privacy or professional discretion. Nonetheless, the evaluation underscores the need for further research on how privacy expectations, consent practices, and access rights can be harmonized across diverse organizational settings.

### Operationalizing information governance and data management

A central objective of the framework is to embed IG/DM principles into the architecture rather than treat them as external policies. EVAL 2 indicated that the architecture consistently enforced consent verification, audit logging, and read‑only, source‑controlled access during the scenario walkthrough. These outcomes show that governance requirements can be operationalized within the interaction flow itself. By implementing HIQ constraints (R6‑R7) across layers, the architecture ensures that information surfaced during coordination is accurate, current, consistent, complete, readable, and contextually relevant. This integration of IG/DM and HIQ strengthens trustworthiness and reduces the risk of unintended disclosure or misuse when information is shared and accessed across organizational boundaries.

### Stakeholder dynamics and pathway complexity

The evaluation highlighted the dynamic and evolving constellation of stakeholders involved in multimorbid care. Beyond the stakeholders captured in Table 2, additional actors, such as social welfare services, police, prison health services, and pathway coordinators, may participate in patient pathways. This variability reflects the inherently fluid and unpredictable nature of inter-organizational clinical work [15–17, 22, 35], and especially for multimorbid patients with unpredictable, non-linear pathways, as illustrated in the user scenario. Anticipated influential future stakeholders include security and IG/DM experts (e.g., super users, data stewards, information architects), system vendors, and ICT services at the hospital, regional, and national levels. By focusing on patient-centric pathways rather than organizational silos, the framework remains flexible and can accommodate changes in actors, responsibilities, and communication flows. Ensuring that the patient’s voice, preferences, and contextual circumstances remain visible across these transitions may help reduce the current imbalance in information logistics observed in the scenario and mitigate risks associated with compartmentalization (F6).

### Integration of emerging technologies

Building on the framework’s ability to accommodate diverse stakeholders and evolving pathway structures, it is also important to consider how emerging technologies can be integrated without disrupting governance, workflow alignment, or information quality. The present study does not describe any specific emerging technologies, such as blockchain or AI components. However, given its layered structure, modular design, and information-centric approach based on information tokens, it is relevant to consider how such technologies could be incorporated as future extensions to the architecture. For example, information tokens could be persisted within a blockchain or similar distributed ledger technologies in the bottom layer’s metadata storage component to strengthen trust and traceability [62]. In contrast, support for AI‑enhanced filtering or summarization must be addressed in future work, particularly in light of the present focus on clinicians’ information exploration.

The vertical pillars, together with provenance and consent checking, must ensure that any future ICT components operate within the same constraints that sustain safety, transparency, and trust across organizations. However, it should be noted that any new technology needs to be considered within the broader socio-technical context. Especially for emerging technologies, additional considerations can arise, such as performance and energy use [62, 63], and AI governance to manage the risks of hallucinations and bias associated with pre-trained AI models [61].

### Extending design-knowledge on inter-organizational information logistics

Related work has shown that interoperability efforts, whether based on national infrastructures, patient-facing portals, or international standards, have improved technical connectivity but still struggle to support pathway‑driven information use [29–34]. Prior solutions often lack structural mechanisms for maintaining HIQ, trust, and provenance across organizations, relying instead on messaging workflows or record replication. The present study contributes design‑oriented knowledge by offering an information‑centric architecture that embeds governance, quality, and coordination into its structural form. EVAL 2 demonstrated that functional requirements (R1–R7) were enacted within the architectural boundaries created by the non‑functional constraints. The framework, therefore, complements existing approaches by specifying how pathway‑oriented information logistics can be structurally supported through information tokens, layered decomposition, and governance‑aligned interfaces. As a conceptual Meta‑artifact, the framework provides a reusable template for future instantiation and inter‑organizational integration initiatives.

### Limitations

This study presents an early-stage conceptual Meta-artifact, and its findings should be interpreted with this maturity in mind. The framework has not yet been technically instantiated or tested in operational settings, which means that empirical evidence regarding performance, usability, workflow integration, and system-level interoperability remains unavailable. These aspects must be examined through future prototyping and implementation studies to reveal how the architecture performs under real conditions, including variable workloads, latency, and local governance routines.

The empirical grounding for the problem analysis was based on a synthesized review of medical records from 14 multimorbid patients. While this provided a rich and diverse dataset spanning primary and specialist care, the sample reflects only a subset of multimorbidity pathways within a specific Norwegian context. The patterns of information fragmentation, documentation variability, and coordination challenges revealed in this cohort are indicative but cannot capture the full diversity of clinical pathways, professional practices, or infrastructural differences present in other regions. Although the architecture is designed to be standards-neutral and theoretically applicable beyond this context, successful adaptation will depend on alignment with local infrastructures, governance arrangements, and regulatory frameworks.

Another limitation concerns the scope of stakeholder involvement. This stage of the research did not include structured participation from patients, frontline clinicians, nurses, system vendors, or data-governance actors. As the analysis showed, information logistics failures are deeply intertwined with workflow routines, professional expectations, trust relationships, and local interpretations of responsibility. These socio-technical dimensions cannot be fully addressed through document analysis alone and require iterative, human-centered evaluation in subsequent DSR cycles. Broader engagement is essential to refine requirements, test usability, and ensure that the framework supports the practical realities of inter-organizational care.

The framework introduces governance-oriented mechanisms such as consent verification, audit logging, and HIQ-based quality constraints. How these controls operate in practice and how consistently they can be implemented across organizational boundaries remain open questions. Opening access to distributed information introduces risks, including unintended disclosures, inconsistencies across documentation cultures, and tensions between transparency and professional discretion. Ensuring that consent handling, data-quality routines, and access controls function harmoniously across diverse providers will require empirical validation.

While the architecture may be extended in future work to incorporate emerging technologies, the extent to which such integrations can be realized in practice remains uncertain. This includes considerations related to computational load, energy requirements, governance of algorithmic tools, and the need to mitigate bias or hallucinations in AI-generated outputs. These uncertainties must be examined carefully before such approaches can be responsibly adopted in inter-organizational clinical settings.

Finally, while the collaboration of researchers from clinical, sociological, and technical backgrounds strengthens the internal validity of the analysis, the absence of broader stakeholder involvement limits the completeness of the requirements and the evaluation of the artifact. Future work should incorporate longitudinal and participatory evaluations to examine how the architecture influences cognitive workload, response time, coordination practices, and the integration of the patient’s voice across pathway transitions.

These limitations collectively point to the need for further DSR iterations, including prototype development, stakeholder-driven refinement, socio-technical evaluation in live environments, and exploration of how the framework can be harmonized with the governance and infrastructural conditions of different healthcare systems.

### Future research directions

To advance the utility of this prescriptive framework and guide its transition from a conceptual architecture to technical instantiation, future research should prioritize the following directions:

1. Inter-organizational IG/DM and behavioral complexity: Further work is needed to examine how inter-organizational governance and data-management structures can be implemented at scale. This includes exploring how governance routines, data quality practices, and consent management workflows can be harmonized across heterogeneous providers. Particular attention should be paid to the sources of behavioral complexity that arise from diverse documentation cultures, professional norms, and trust relationships. Research should also investigate how architectural mechanisms such as tokens, provenance, and audit trails can be aligned with organizational incentives to support sustained HIQ and responsible local stewardship.
2. Component prototyping to support clinical reasoning: Building on the middle-layer components of the framework, future studies should develop and test prototype modules that support both rapid, intuitive sense-making and more detailed analytical review. This includes investigating how semantic enrichment, contextual filtering, and adaptive navigation can improve clinicians’ time-to-answer and reduce cognitive load during cross-organizational decision-making.
3. Governance of intelligent information access: As technologies such as AI-assisted summarization, semantic mapping, or advanced filtering are emerging, research must examine how these tools can operate within the architecture’s IG/DM and HIQ constraints. Such work should evaluate how data-driven services can be made transparent, controllable, and safe, and how their outputs can be aligned with FATE principles (Fairness, Accountability, Transparency, and Ethics) to avoid bias, hallucination, and other unintended consequences.
4. Longitudinal human-centered evaluation: A key next step is the empirical assessment of the architecture through interactive prototypes in clinical environments. Such studies should examine how the framework influences information logistics in practice, including its effect on cognitive workload, coordination efficiency, patient involvement, and the quality and timeliness of information flows. Longitudinal and participatory evaluation will be crucial for refining the artifact and understanding its suitability across diverse organizational contexts.

These research directions collectively outline how the conceptual framework can be extended, validated, and adapted through successive DSR iterations, ultimately informing future implementation efforts within inter-organizational health information environments.

The Valkyrie project will continue advancing these architectural dimensions through iterative prototyping of the virtual EHR and patient-centric pathway models and will report on this work as it evolves in future publications.

## Conclusions

This study set out to address persistent gaps in inter-organizational information logistics by developing a conceptual, information-centric architecture framework grounded in real-world clinical complexity. Guided by the Design Science Research paradigm, the framework synthesizes insights from empirical analyses of multimorbid patient trajectories, socio-technical theory, interoperability literature, and established systems engineering principles. The resulting Meta-artifact provides a prescriptive blueprint that seeks to mitigate two fundamental challenges identified in the problem space: insufficient awareness of parallel treatment activities and limited access to distributed patient records.

The architecture responds to these challenges through two core mechanisms. First, it supports proactive awareness by enabling context-sensitive notifications of concurrent clinical events. Second, it introduces governed virtual access to distributed records through information tokens and read-only retrieval, avoiding the quality risks associated with record replication. These mechanisms are anchored in the framework’s vertical pillars, Security and Privacy, Information Governance, Data Management, and Health Information Quality, which collectively establish the conditions needed for trustworthy, pathway-oriented information use across organizations.

As an early-stage conceptual contribution, the framework does not claim to provide empirical validation or performance evidence. Its role is to provide prescriptive design knowledge to inform subsequent iterations of technical development, prototyping, and evaluation in clinical environments. The limitations reflect this stage of maturity: the artifact has not yet been implemented in operational systems, has not undergone stakeholder-based usability testing, and has not been evaluated under real-world workload, latency, or governance conditions. Further research should focus on the instantiation of key components, the assessment of cognitive and workflow impacts, and the exploration of how the architecture can be integrated into existing infrastructures without disrupting established workflow and routines.

Nonetheless, by articulating a theoretically grounded and empirically informed information architecture framework, this study contributes a structured basis for advancing inter-organizational information logistics. It offers a clear direction for future development efforts and provides a foundation for addressing the socio-technical and infrastructural challenges that continue to impede integrated care.

## Supplementary Information

Below is the link to the electronic supplementary material.

Supplementary Material 1

## Data Availability

The detailed scenario description is available as a supplement to this manuscript. However, the data used to construct the scenario cannot be shared due to its sensitive nature and the strict privacy regulations governing patient records in Norway.

## Abbreviations

AI: Artificial Intelligence
CNS: Community Nursing Service
DM: Data Management
DSR: Design Science Research
EHR: Electronic Health Records
FHIR: Fast Healthcare Interoperability Resources
GP: General Practitioner
HIQ: Health Information Quality
HL7: Health Level Seven
IG: Information Governance
IHE: Integrating the Healthcare Enterprise
ISO: The International Organization for Standardization
UC: Urgent Care
