# Supplementary material for: Towards an information-centric architecture framework for health information logistics: a design science research study
Source: BMC Med Inform Decis Mak. 2026 Jun 26;26:347. doi: 10.1186/s12911-026-03642-7 (PMC13573431; doi:10.1186/s12911-026-03642-7)
Supplement: Supplementary file 1 [file 12911_2026_3642_MOESM1_ESM.docx]

**Supplemental Digital Appendix (SDA)**

**Appendix A**

#### User Scenario – Parallel Clinical Pathways

*In the spring of 2005, the patient was recorded in the hospital's EHR system due to a defective heart valve, necessitating the implantation of a mechanical prosthetic heart valve. After surgery, the patient attended regular follow-ups to monitor valve function and manage anticoagulation therapy until 2024. The black line symbolizes this clinical pathway as a continuous sequence of actions.*

*Shortly after heart surgery, the patient experienced facial asymmetry and speech difficulties (red line). In 2006, a cerebral aneurysm was diagnosed, requiring neurosurgical intervention and speech therapy. Later that year, mild cognitive impairment was documented. Although no further events related to this condition appeared after 2006, the red line symbolizes its chronic nature. Between 2008 and 2017, aside from routine heart valve monitoring and anticoagulation adjustments, no additional events were recorded. In early 2018, visual disturbances and headaches emerged, leading to a diagnosis of retinal vascular occlusion by early 2019 (green line). Subsequent follow-ups focused on status evaluations, with no disease progression.*

*In August 2022, the patient developed depression and suicidal ideation (blue line). For the first time, the GP referred the patient to a psychiatric hospital. A major depressive disorder was diagnosed in November 2022. Concurrently, the patient reported somatic symptoms such as abdominal pain, chest discomfort, dyspnea, lower back pain, hematochezia, and muscle cramps. The GP referred the patient twice to Cardiology, with the second referral noting depressive episode codes, and to Gastroenterology via UC for persistent abdominal pain. A review revealed two prior referrals in 2019 to Internal Medicine and Cardiology for gastrointestinal and back pain complaints, associated with congenital aortic valve disease (black line) and diarrhea episodes.*

*In November 2023, the patient recovered after hospitalization for COVID-19, delirium, and a depressive episode, requiring multidisciplinary care involving medical and psychiatric teams. The patient was discharged to live with a family member. During this period, the family member’s caregiving capacity declined, prompting increased coordination between the GP, CNS, and family members to arrange home care or institutional care. Documentation of the patient’s voice and input from next of kin appeared in the EHR, although this information remained compartmentalized.*

#### User Scenario – the Convergence of the Clinical Pathways

*At the beginning of 2024, the patient stayed temporarily at a community care institution and was admitted to the hospital with tubulointerstitial nephritis and sepsis. During this hospitalization and the following months, clinical documentation increasingly combined codes related to mental and physical conditions, illustrated with circles including all four colors (pathways).*

*On January 16, the hospital’s medical department advised the GP and CNS to arrange for institutional or home care. Three days later, the patient was transferred to community care, arriving unstable, confused, and missing medication and an updated medication list.*

*On January 22, the GP requested the hospital’s medication list and details of an upcoming appointment, and also asked CNS about plans for food delivery at home. On January 23, CNS messaged the GP, concerned about missing medication and whether suicidal intent was a real threat. The next day, the GP reported the patient being calmer but needed more practical help and permanent housing closer to the city.*

*On January 26, the GP agreed with the nurse to administer medication. Two days later, CNS informed the GP that the patient refused nurse-administered medications. The GP then requested a coordination meeting between the patient, next of kin, CNS, and GP. After the meeting on February 7, the GP referred the patient to the hospital's psychiatric department. The hospital denied the referral on February 20 due to ongoing examination at various institutions and an unstable family and housing situation. On March 14, the GP sent a new referral, which was denied on March 18 for the same reason.*
